# Genetic Structure and Selection of a Core Collection for Long Term Conservation of Avocado in Mexico

**DOI:** 10.3389/fpls.2017.00243

**Published:** 2017-02-24

**Authors:** Luis F. Guzmán, Ryoko Machida-Hirano, Ernesto Borrayo, Moisés Cortés-Cruz, María del Carmen Espíndola-Barquera, Elena Heredia García

**Affiliations:** ^1^Centro Nacional de Recursos Genéticos, Instituto Nacional de Investigaciones Forestales, Agrícolas y PecuariasTepatitlán de Morelos, Mexico; ^2^Gene Research Center, University of TsukubaTsukuba, Japan; ^3^Fundación Salvador Sánchez Colín, CICTAMEX S. C., Ignacio Zaragoza, Col. CentroCoatepec Harinas, Mexico; ^4^Campo Experimental Bajío, Instituto Nacional de Investigaciones Forestales, Agrícolas y PecuariasCelaya, Mexico

**Keywords:** *Persea americana*, microsatellites, botanical races, core collection, PCA-Kmeans

## Abstract

Mexico, as the center of origin of avocado (*Persea americama* Mill.), harbors a wide genetic diversity of this species, whose identification may provide the grounds to not only understand its unique population structure and domestication history, but also inform the efforts aimed at its conservation. Although molecular characterization of cultivated avocado germplasm has been studied by several research groups, this had not been the case in Mexico. In order to elucidate the genetic structure of avocado in Mexico and the sustainable use of its genetic resources, 318 avocado accessions conserved in the germplasm collection in the National Avocado Genebank were analyzed using 28 markers [9 expressed sequence tag-Simple Sequence Repeats (SSRs) and 19 genomic SSRs]. Deviation from Hardy Weinberg Equilibrium and high inter-locus linkage disequilibrium were observed especially in *drymifolia*, and *guatemalensis*. Total averages of the observed and expected heterozygosity were 0.59 and 0.75, respectively. Although clear genetic differentiation was not observed among 3 botanical races: *americana, drymifolia*, and *guatemalensis*, the analyzed Mexican population can be classified into two groups that correspond to two different ecological regions. We developed a core-collection by K-means clustering method. The selected 36 individuals as core-collection successfully represented more than 80% of total alleles and showed heterozygosity values equal to or higher than those of the original collection, despite its constituting slightly more than 10% of the latter. Accessions selected as members of the core collection have now become candidates to be introduced in cryopreservation implying a minimum loss of genetic diversity and a back-up for existing field collections of such important genetic resources.

## Introduction

Mexican avocado (*Persea americana* Mill.) has been insufficiently studied in its own center of origin despite the wide genetic diversity the region hosts. Understanding its unique population structure and domestication history, as well as exploring strategies for its conservation, has been a long-neglected priority.

Mexico is the world's largest producer of avocados, representing over one-third of global production and harvested area. Avocado is one of the most economically important fruit trees to Mexico and Central America. It is an evergreen subtropical species that has adapted to different climate ranges, extending its production to North and South America, the Caribbean, Asia, Africa, Middle East, and Europe (Food Agriculture Organization of the United Nations, 2014 WEB reference). Such geographical outspread implies adaptation to each specific local environment, which could derive into a reduction of genetic variation due to isolation of populations.

Avocado is a member of a large family called Lauraceae, constituted by 50 genera, including the genus *Persea*. There are three botanical races: (1) *P. americana* var. *drymifolia* (Schlecht. & Cham.) Blake (Mexican race), (2) *P. americana* var. *guatemalensis* L. Wms. (Guatemalan race), and (3) *P. americana* var. *americana* Mill. (West Indian race) (Table 1). They are recognized as commonly cultivated avocadoes (Ashworth et al., 2011) and their hybrids are currently cultivated worldwide (Borrone et al., 2009). These botanical races are determined by morphological, geographical, physiological, biochemical, molecular, and commercial aspects although some experts have proposed that differences among them are unclear (Ashworth et al., 2011). In addition, within *P*. *americana*, other taxa—var. *nubigena* (L. O. Williams) Kopp (1966) and var. *costaricencis* Ben-Ya'acob et al. (2003)—are also recognized.

**Table 1:** **Summary of main characteristics of botanical races of cultivated avocado**.

**Taxon**	**Botanical race**	**Key characteristics**	**Climatic adaptation**	**Distribution range and putative origin**
*P. americana* var. *drymifolia* (Schltdl. & Cham.) Blake	Mexican race	Fruit is 4–12 cm long; fruit skin is very thin with a purple-to-black, dark-green hue; leaves and flesh have an anise-like scent; flesh oil content is high.	Subtropical, moderate to high cold tolerance.	Central highland regions in Mexico.
*P. americana* var. *guatemalensis* L. Wms.	Guatemalan race	Fruit is 10–18 cm long; fruit skin is thick, often woody and brittle with a rough surface; fruit skin is green or black; generally small seed tight in cavity; flesh oil content is moderately high.	Subtropical, moderate cold tolerance.	Guatemalan mountain tracts and lowlands.
*P. americana* var. *americana* Mill.	West Indian race	Fruit is 10–25 cm long; fruit skin is thin to moderately thin with a pale green through maroon hue; flesh oil content is low.	Tropical range, low cold tolerance.	Hot and humid lowlands from Guatemala through Costa Rica and northern South America.

*Scora et al., 2002; Ashworth et al., 2011; Ayala and Ledesma, 2014*.

According to archeological evidence, avocado consumption began more than 7,000 years ago (Smith, 1966) and it has been considered as one of the trees to be domesticated previous to annual crops in Mesoamerica (Galindo-Tovar et al., 2008). Furthermore, ancient ethnic groups may have contributed to some extent to the divergence of avocado into different botanical races through selection of the variants that best adapted to different environmental conditions (Cañas-Gutiérrez et al., 2015). Founded on genetic markers, there is a wide variety of studies regarding the genetic diversity and domestication history of avocado (AFLP's, Cañas-Gutiérrez et al., 2015; RAPD's, Sharon et al., 1997; RFLP's and VNTR's, Chanderbali et al., 2008; SNP's, Chen et al., 2008, 2009). Among these, microsatellites are commonly used for genetic diversity studies due to their reproducibility, high variability, codominance inheritance, abundance, and distribution across the avocado genome (Ashworth et al., 2004; Gross-German and Viruel, 2013). Although genetic diversity in wild avocado varieties has been reported (Chen et al., 2008), this is not the case for an extensive genetic diversity evaluation and phylogenetic relationship that may focus specifically in the avocado germplasm present in Mexico.

Genetic diversity plays an important role not only in preservation of biodiversity, but also of ecological and cultural aspects, such as diet and economy (Sarr et al., 2008; Rincón-Hernández et al., 2011). Projects have been launched with the aim to avoid the loss of such important resources. The conservation of plant species depends greatly on whether their seeds are of orthodox or recalcitrant nature. The latter species are often conserved in field collections, as is the case for the local collection in Celaya Experimental Station of the National Forestry, Crops, and Livestock Research Institute (CEBAJ-INIFAP), which dates back to 1972 and whose main objective is to collect and conserve varieties of avocado local to Mexico. At present time, this collection has been used as a source for cultivation and breeding materials retrieval. Recently, an initiative has arisen for the establishment of the “National Avocado Germplasm Depository (BNGA),” under a joint effort between the Salvador Sanchez Colin Foundation, CICTAMEX and CEBAJ-INIFAP. The collection—consisting of more than 350 accessions, mainly of Mexican genotypes (*P. americana* var. *drymifolia*) collected from different localities across Mexico—aims to conserve, as backup, these accessions in different institutions.

For the long-term conservation and/or safety backup of field collection of recalcitrant species, as avocado is, two main approaches are usually taken: *in vitro* and cryopreservation (Efendi and Litz, 2003). Specific protocols for target species must be available for these approaches to be successful. In addition, preparation and maintenance of *in vitro* cultures require skilled technicians and a maintenance budget. Cryopreservation has been recognized as the safest alternative for long-term conservation of plant genetic resources, as it does not require continuous manipulations (González-Arnao et al., 2014). To ensure the conservation and sustainable utilization of recalcitrant species under limited resources, we propose: a combination of cryopreservation with Core-Collection (CC) selection, i.e., a reduction to a small-number subset of the larger germplasm collection representing the maximum possible genetic diversity with minimum repetitiveness (Frankel, 1984). The CC represents an ideal set of accessions to use for the establishment of cryopreservation protocols and for the initial introduction of accessions in cryostorage. The combination of CC and cryopreservation approaches will ensure a feasible long-term conservation of avocado by focusing on a limited number of representative elements with the safest conservation method.

In this study, we carried out a genetic diversity evaluation of avocado germplasm conserved in the CEBAJ-INIFAP genebank by means of microsatellite markers, in order to infer variation and phylogenetic relationships which will in turn provide the relevant information to deduce the domestication history of avocado and to identify a CC that contained a representative subset -in terms of genetic diversity- of the original collection to ensure efficient long-term germplasm preservation.

## Materials and methods

### Plant material and genomic DNA extraction

A total of 319 accessions were sampled at Campo Experimental Bajío, INIFAP, in Mexico. The collection includes 313 of *Persea americana*. Among these, information on botanical races [var. d*rymifolia* (104), var. *guatemalensis* (15), var. *americana* (12), var. *costareicensis* (3), and hybrids (31)] was available for 165 accessions, while information about geographical origin was available for 299 accessions. Three other *Persea* species [*P*. *longipes* (2), *P*. *nubigena* (2), and *P*. *schiedeana* (1) and an accession from other genus (*Beilmiedia anay*)] are also included in the collection. Fresh leaves collected from individual trees in the field were transferred to the laboratory and freeze-dried on the same day. Total DNA was extracted by a modified tropical-plant-specific protocol (Huang et al., 2013) to overcome polysaccharide and phenolic compound complications. The extraction buffer contained 2 M of NaCl, 25 mM of ethylenediaminetetraacetic acid, 200 mM of Tris, 2% of cetyltrimethylammonium bromide, 2% of polyvinylpolypyrrolidone, 1% of lauroyl sarcosine, 20 mM of borax, and 140 mM of β-mercaptorthanol. During incubation (65°C for 45 min), 700 μl of dichloromethane was used instead of the original-protocol chloroform: phenol: isoamyl alcohol (25:24:1). Incubation and precipitation were performed twice, followed by the previously reported unmodified protocol.

### Microsatellite analysis

A total of 47 EST-SSR and the genomic SSR (Gross-German and Viruel, 2013) were tested using an aleatory generated number (4) of genotypes randomly selected from the BNGA collection. Within all the 47 sets of primers, we discarded primer pairs which produced an ambiguous allelic pattern, and either monomorphic or more than two alleles. Finally, 28 primer pairs (9 EST-SSR and 19 genomic SSR) were used for genotyping. A combination of Universal tailed primers with multiplex PCR approach was applied for genotyping. A specific DNA sequence was attached to the 5′ end of each forward primer (tailed forward primer, Blacket et al., 2012). For microsatellite amplification, combinations of a tailed forward primer; a tail primer labeled with one of 6-FAM, VIC, PET, or NED (Life Technologies) dyes consisting of the complementary tail sequence; and a reverse primer were used. The multiplex PCR reactions were performed with four different marker sets in a total volume of 10 μL containing approximately 10 ng of template DNA using 1 × QIAGEN Type-it Microsatellite PCR Kit (QIAGEN) according to the manufacturer's standard protocol. PCR cycles consisted of a denaturing step of 15 min at 95°C; followed by 30 cycles of 94°C for 30 s, 57°C for 90 s, and 72°C for 60 s; and a final elongation step of 30 min at 60°C. PCR products were determined by capillary sequencer (3500xl Genetic Analyzer, Life Technologies). The allelic composition of each marker was determined for each accession, and putative alleles were indicated by the estimated size in base pair by GeneMapper (Life Technologies).

### Genetic diversity analysis

A total of 319 accessions of the BNGA collection were genotyped. One accession was not included due to low yield of amplicon. The botanical race information on the 165 accessions had been previously determined by a genebank curator regarding the fruit-skin characteristics and anise-like odor of the leaf. Number of total alleles scored (*A*), observed heterozygosity (*H*o), unbiased expected heterozygosity (u*H*e), and fixation index (*F*) were calculated to determine the genetic diversity and the past inbreeding. Allele frequencies of each botanical race and deviation from Hardy-Weinberg equilibrium (HWE) of loci were calculated for the subset of genotyping data on 165 accessions with botanical race information. All these values were calculated using GenAlEx 6.5 (Peakall and Smouse, 2012). Allelic richness (A_R_) and private allelic richness (PA_R_) for each botanical race were measured via rarefaction by HP-Rare v.1.0 (Kalinowski, 2005), calculated based on a minimum sample size of 12 (sample number of *americana*). Linkage disequilibrium (LD) between pairs of markers was tested by Genpop on the Web (Raymond and Rousset, 1995; Rousset, 2008; http://genepop.curtin.edu.au/).

### Race assignment and genetic group classification of *P. americana* in BNGA

The membership compositions of each cluster, inferred by STRUCTURE analysis (Pritchard et al., 2000), were compared with the subset of 165 accessions with botanical race classification defined by morphological characteristics. The analysis was carried out to infer the most-likely number of genetic clusters (*K*) for the whole set of accessions showing both admixture and non-mixture by correlated allele frequency models.

Firstly, we screened numbers of clusters (*K*) from 1 to 8; each *K* was simulated 5 times with a burn-in of 10,000 iterations before collecting data, and 10,000 iterations of the Markov-Chain Monte Carlo (MCMC) method. Secondly, to obtain accurate results, parameter estimates were increased to a burn-in of 100,000 iterations and 100,000 iterations of MCMC with K from 1 to 5 with 5 iterations. The optimum *K* value was determined from log probability of data at each step of the MCMC, Pr(X|*K*), and *ad hoc* statistic Δ*K* of Evanno et al. (2005) using STRUCTURE HARVESTER (Earl and von Holdt, 2012). Genetic groups determined by STRUCTURE were then projected on a map using data from the 299 accessions with information on geographical origin. Geographical coordinate data was obtained from passport data, either as a georeferenced position or based on the localities of collection. For accessions that only had country of origin, the information was represented as the capital city's geographical coordinates.

### Selection and validation of core-collection

PCA-Kmeans method (Borrayo et al., 2016) was used for CC selection. The CC is intended to select a small subset of individuals of the National Avocado Germplasm Depository (BNGA) for long-term conservation by *in vitro* culture and cryopreservation. Only genotyping data were used as input in order to retain as much genetic variation as possible. We arbitrarily set the CC-number to 36 accessions (12.1% of the original collection), in what we consider to be a manageable sub-collection and in agreement with previous reports that establish that CC should contain ≥10% elements from the original collection (Guo et al., 2014), and then evaluated along with different CC sizes: 12, 24, 48, 72, and 96. Input data were depurated by the elimination of monomorphic alleles and accessions that lacked complete information. Twenty accessions were eliminated due to missing data from a total of 318 accessions genotyped, resulting in 298 accessions with complete genotyping data that served as the original collection for the CC selection procedure. Subsequently, the genotyping matrix with fragment sizes was transformed into a 0-to-1 scale and the PCA-Kmeans CC selection method was applied to the new data matrix. All CC selections and mathematical evaluations were performed with CorColv2.1-beta (program is available on request), an MS-Windows executable file built from a Python implementation of original FREEMAT codes published by Borrayo et al. (2016). A phenetic tree was constructed based on the same data using the neighbor-joining method (Saitou and Nei, 1987) in PowerMarker 3.25 (Liu and Muse, 2005) with 1,000 bootstrap replications. Distributions of CC members on the dendrogram were visualized by MEGA 5.2 (Tamura et al., 2011). The selected CC was validated by both mathematical evaluation parameters and their distribution patterns on the dendrogram; as well as by race information, geographical origin, and ratio of genetic group determined by STRUCTURE analysis.

## Results

### Genetic diversity of BNGA collection

The 28 markers that were analyzed (9 EST-SSR and 19 SSRs) detected a total of 547 alleles ranging from 4 to 34 alleles with an average of 19.1 per locus, out of which, 393 (71.7%) were rare alleles present in frequency less than 0.05. Except for LMAV20, all the markers tested were not under HWE (Table 2). Deviations from HWE of each marker were also tested by botanical race groups. Twenty-seven loci were not in HWD in *drymifolia*, 16 in *guatemalensis*, one in *americana*, and none in *constericensis* (Table 3). Out of 378 observed marker combinations, we detected significant (*p* < 0.01) LD between pairs of markers in 344 (91.0%) combinations in all 318 accessions. The botanical race-wise values for significant LD were 205 (54.2%), 125 (33.1%), and 5 (1.3%) combinations for *drymifolia, guatemalensis*, and *americana*, respectively. For *costaricensis*, only six marker combinations were valid to calculate LD due to the small number of samples, none of which was significant.

**Table 2:** **Diversity parameters associated with the 318 accessions of avocado analyzed**.

**Locus name**	**Na**	**Ne**	***H*o**	***H*e**	***F***	**HWE all**	**HWE CC36**
ESTAVAG28	13	1.99	0.38	0.50	0.24	^***^	^***^
ESTAVAG31	24	6.34	0.74	0.84	0.12	^***^	^***^
ESTAVGA01	12	3.17	0.53	0.68	0.23	^***^	^***^
ESTAVGA03	21	8.09	0.69	0.88	0.21	^***^	^**^
ESTAVTA02	12	5.99	0.69	0.83	0.18	^***^	^**^
ESTAVTC03	24	9.78	0.64	0.90	0.29	^***^	^***^
ESTAVTC13	24	8.29	0.72	0.88	0.18	^***^	^***^
ESTAVTC18	33	7.51	0.81	0.87	0.06	^***^	^*^
ESTAVTC20	21	10.65	0.70	0.91	0.23	^***^	^***^
Average est-SSRs	20.44	6.87	0.65	0.81	0.19		
LMAV01	24	7.00	0.60	0.86	0.30	^***^	^***^
LMAV06	26	8.39	0.77	0.88	0.13	^***^	^***^
LMAV08	16	7.85	0.75	0.87	0.14	^***^	^*^
LMAV13	17	5.20	0.70	0.81	0.13	^***^	^**^
LMAV14	23	4.79	0.63	0.79	0.21	^***^	^***^
LMAV15	20	7.78	0.73	0.87	0.16	^***^	ns
LMAV16	17	2.83	0.42	0.65	0.35	^***^	^***^
LMAV18	27	9.01	0.83	0.89	0.07	^***^	^*^
LMAV19	34	11.68	0.73	0.91	0.20	^***^	ns
LMAV20	4	1.07	0.05	0.06	0.18	^*^	^**^
LMAV24	16	5.70	0.69	0.82	0.16	^***^	^***^
LMAV25	13	6.36	0.62	0.84	0.26	^***^	^***^
LMAV26	15	2.98	0.49	0.66	0.27	^***^	ns
LMAV27	8	3.32	0.57	0.70	0.18	^***^	^***^
LMAV29	25	5.04	0.60	0.80	0.25	^***^	^***^
LMAV30	19	2.66	0.28	0.62	0.55	^***^	^***^
LMAV31	33	16.83	0.79	0.94	0.16	^***^	^*^
LMAV33	14	5.51	0.64	0.82	0.22	^***^	^***^
LMAV34	12	3.94	0.31	0.75	0.59	^***^	^***^
Average SSRs	19.1	6.21	0.59	0.77	0.24		
Total average	19.5	6.42	0.61	0.78	0.22		
CC36 average	12.1	6.26	0.60	0.75	0.22		

*** *p < 0.001*;

** *p < 0.01*;

* *p < 0.05; ns, not significant*.

**Table 3:** **Result of HWE statistics of each botanical race**.

**Locus**	***drymifolia*** **(*n* = 104)**				***guatemalensis*** **(*n* = 15)**				***americana*** **(*n* = 12)**				***costaricensis*** **(*n* = 3)**			
	**DF**	**ChiSq**	**Prob**	**Signif**	**DF**	**ChiSq**	**Prob**	**Signif**	**DF**	**ChiSq**	**Prob**	**Signif**	**DF**	**ChiSq**	**Prob**	**Signif**
ESTAVAG28	55	198.835	0.000	^***^	15	52.322	0.000	^***^	21	27.480	0.156	ns	3	3.333	0.343	ns
ESTAVAG31	136	264.762	0.000	^***^	36	72.292	0.000	^***^	45	50.684	0.259	ns	6	4.500	0.609	ns
ESTAVGA01	36	73.779	0.000	^***^	10	34.792	0.000	^***^	10	11.648	0.309	ns	3	3.333	0.343	ns
ESTAVGA03	210	439.829	0.000	^***^	66	83.786	0.069	ns	45	63.667	0.035	^*^	6	6.000	0.423	ns
ESTAVTA02	66	159.740	0.000	^***^	28	27.350	0.499	ns	21	34.814	0.030	^*^	3	3.333	0.343	ns
ESTAVTC03	153	481.588	0.000	^***^	78	121.250	0.001	^**^	36	70.000	0.001	^***^	6	6.000	0.423	ns
ESTAVTC13	171	430.486	0.000	^***^	36	61.458	0.005	^**^	36	50.880	0.051	ns	3	6.000	0.112	ns
ESTAVTC18	378	587.332	0.000	^***^	91	118.333	0.029	^*^	45	47.253	0.381	ns	10	12.000	0.285	ns
ESTAVTC20	171	355.606	0.000	^***^	36	78.646	0.000	^***^	55	82.167	0.010	^*^	10	12.000	0.285	ns
LMAV01	120	401.905	0.000	^***^	21	40.807	0.006	^**^	36	48.480	0.080	ns	10	9.000	0.532	ns
LMAV06	210	644.523	0.000	^***^	55	98.067	0.000	^***^	78	70.653	0.710	ns	3	3.333	0.343	ns
LMAV08	105	355.366	0.000	^***^	36	36.788	0.432	ns	45	53.040	0.192	ns	6	9.000	0.174	ns
LMAV13	91	154.598	0.000	^***^	28	50.934	0.005	^**^	21	30.013	0.092	ns	3	6.000	0.112	ns
LMAV14	136	427.146	0.000	^***^	45	72.083	0.006	^**^	45	50.204	0.275	ns	3	0.750	0.861	ns
LMAV15	105	216.409	0.000	^***^	55	92.929	0.001	^**^	45	42.933	0.560	ns	15	15.000	0.451	ns
LMAV16	78	382.322	0.000	^***^	36	79.157	0.000	^***^	28	33.524	0.217	ns	6	9.000	0.174	ns
LMAV18	210	392.078	0.000	^***^	66	91.417	0.021	^*^	28	21.500	0.804	ns	6	9.000	0.174	ns
LMAV19	351	843.058	0.000	^***^	120	162.604	0.006	^**^	36	42.720	0.205	ns	3	3.333	0.343	ns
LMAV20	3	3.671	0.299	ns	1	0.018	0.894	ns	1	0.023	0.880	ns	1	0.333	0.564	ns
LMAV24	78	309.163	0.000	^***^	28	32.698	0.247	ns	45	48.000	0.352	ns	10	12.000	0.285	ns
LMAV25	78	393.112	0.000	^***^	36	57.213	0.014	^*^	45	60.667	0.059	ns	6	6.000	0.423	ns
LMAV26	78	205.491	0.000	^***^	21	42.593	0.004	^**^	15	20.474	0.154	ns	1	0.120	0.729	ns
LMAV27	15	49.764	0.000	^***^	10	19.630	0.033	^*^	6	10.698	0.098	ns	6	9.000	0.174	ns
LMAV29	210	558.055	0.000	^***^	66	106.116	0.001	^**^	21	33.083	0.045	^*^	10	9.000	0.532	ns
LMAV30	78	442.789	0.000	^***^	21	68.438	0.000	^***^	10	5.878	0.825	ns	10	12.000	0.285	ns
LMAV31	465	790.086	0.000	^***^	120	149.167	0.037	^*^	153	159.333	0.346	ns	6	6.333	0.387	ns
LMAV33	66	182.449	0.000	^***^	21	33.722	0.039	^*^	36	42.918	0.199	ns	6	6.000	0.423	ns
LMAV34	45	578.061	0.000	^***^	21	54.972	0.000	^***^	10	21.226	0.020	^*^	3	3.333	0.343	ns

*** *p < 0.001*;

** *p < 0.01*;

* *p < 0.05; ns, not significant*.

Analysis of the data subset of 165 accessions with botanical race information provided genetic diversity for each botanical race (Supplementary Figure 1). Values of observed heterozygosity, expected heterozygosity and number of private alleles are shown in Table 4. Allelic richness (A_R_) and private allele richness (PA_R_) in the population were: 5.95 and 0.63 for *drymifolia*, 6.13 and 0.89 for *guatemalensis*, 6.22 and 0.69 for *americana*, and 3.82 and 0.90 for *costaricensis*, respectively (Table 4).

**Table 4:** **Summary statistics for each botanical race**.

**Botanical race**	**n**	***A***	***H*****o**	**u*H*e**	***F***	**A_R_**	**PA_R_**
*drymifolia*	104	2907	0.57	0.74	0.24	5.95	0.63
*guatemalensis*	15	419	0.59	0.78	0.21	6.13	0.89
*americana*	12	334	0.54	0.78	0.27	6.22	0.69
*costaricensis*	3	83	0.57	0.78	0.12	3.82	0.90

*n, sample number; A, total number of allele scored; Ho, observed heterozygosity; uHe, unbiased expected heterozygosity; F, fixation index; A_R_, Allelic richness determined by mean number of alleles per locus; PA_R_, private allele richness determined by frequency of private alleles within populations*.

### Race assignment

Genetic assignment methods implemented in STRUCTURE (Pritchard et al., 2000) revealed that *K* = 2 showed the highest likelihood in Mexican avocado by both the log likelihood [LnP(D)] and the Evanno's Δ*K*. Estimate of population divergence of allele frequencies in these two inferred populations from ancestral frequencies was 0.14 (Cluster A) and 0.05 (Cluster B), respectively.

The values represent the calculated average of 5 repeated runs. Average of a net nucleotide difference between clusters was 0.12. Average distance between individuals was 0.79 (Cluster A) and 0.71 (Cluster B). Accessions were assigned to one of the clusters when more than 80% of the genetic background belonged to such cluster. The number of accessions assigned to each cluster was 71 (22.3%) for Cluster A and 188 (59.1%) for Cluster B. Fifty-nine (18.6%) individuals were considered to be of admixed origin (80> A and B >20) (Supplementary Table 1).

**Figure 1:**
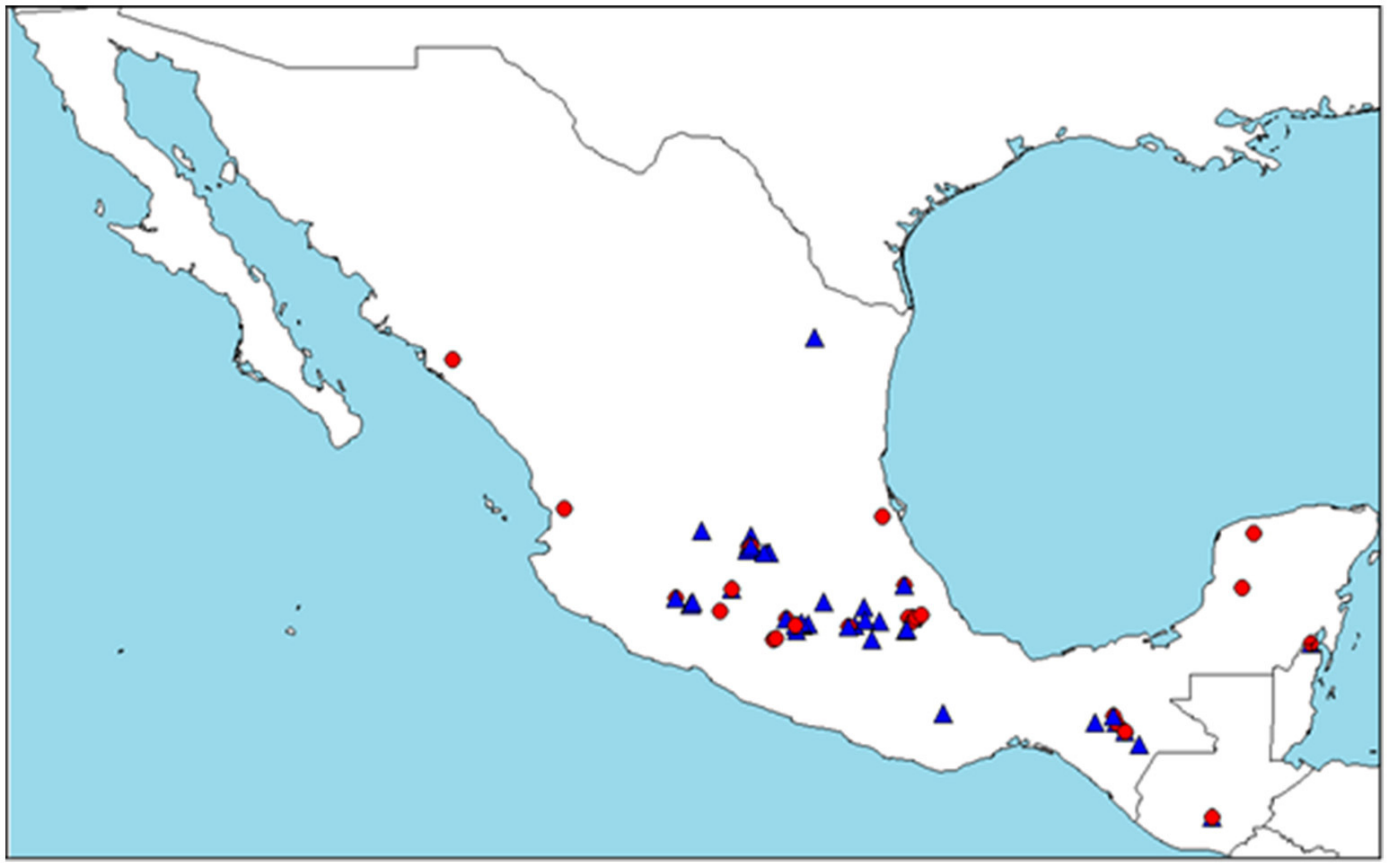
**Geographical distribution of assignment results of STRUCTURE (*K* = 2) analysis**. Only the accessions assigned more than 80% to one of the clusters (A or B) were plotted on the map. The red circle represents Cluster A and the blue triangle, Cluster B. Distribution of the accessions originated in Mexico and Guatemala are shown.

Comparison of these genetic groups assigned by STRUCTURE and their botanical race information did not coincide with each other (Supplementary Figure 2). Therefore, we could not assign the botanical races based on genetic groups determined by STRUCTURE. Precise comparisons were impossible due to the disproportional presence of *drymifolia* compared with other botanical races. Nevertheless, individuals belonging to Cluster B (72) occur about five times more often than those in Cluster A (15) for *drymifolia*. Geographical distribution of Clusters A and B was represented on a map (Figure 1). The distribution of both clusters overlapped with some differences: distribution of Cluster A stretches wider in longitudinal range from the Yucatan Peninsula to the Pacific Coast, whereas distribution of Cluster B is more concentrated in the Central Highland.

### Core collection

As an adequate evaluation threshold was archived at CC36 and above (CC48, 72, and 96), and being 36 the smaller CC to meet this criterion, the core reference set of 36 individuals was selected from the 298 individual accessions. Scores obtained from the evaluation of CC36 were compared with those of other CCs as well as with the original collection (Table 5). In addition to individuals from three botanical races of *P*. *americana*, two other individuals were selected to CC: one belonging to the same sub-genus, *P. americana* var. *nubigena*, and the other from *P. longipes*, which belongs to sub-gender *P. eriodaphne*.

**Table 5:** **Core collection evaluation values for different *K* Core collection sizes**.

***K***	**12**	**24**	**36**
ANE	0.2834	0.2447	0.2192
ENE	0.3138	0.2639	0.3173
E	0.5151	0.5226	0.5833
MD	0.0000	0.0000	0.0000
VD	78.5714	82.1429	67.8571
CR	51.0039	57.7763	72.2067
CV	85.8682	85.8247	97.6196
CA	0.6032	0.7320	0.8103

*K, Elements included in CC; ANE, Average distance between each original collection and the nearest CC sample; ENE, Average distance between each CC sample and nearest CC sample; E, Average distance between CC samples; MD, Homogeneity test for means (mean difference % between CC and the nearest initial collection); VD, Homogeneity test for variance (variance difference % between CC and initial collection); CR, Coincidence rate (CR % retained by the CC is no less than 80%); CV, Variable rate (no more than 20% of the traits have different means between CC and the initial collection); CA, Allele Coverage*.

A smaller ANE (average distance between each original collection and the nearest CC sample) means the diversity in the original collection is homogeneously represented by the CC. On the other hand, ENE (average distance between each CC sample and nearest CC sample) and E (average distance between CC samples) indicate the dispersion of data for the CC, where higher values stand for a better representation of extreme values. Values of ANE decrease as size of the CC increases; this is because members of a larger CC fill the gap of the smaller CC decreasing the average distance between the selected elements of the CC and those of the original collection.

ENE's largest value in CC36 indicates that this CC set is the one with the largest dispersion. The *E*-value increases as the number of CC increases; this indicates that extreme individuals are being included as the extreme values begin to form a single cluster.

**Figure 2:**
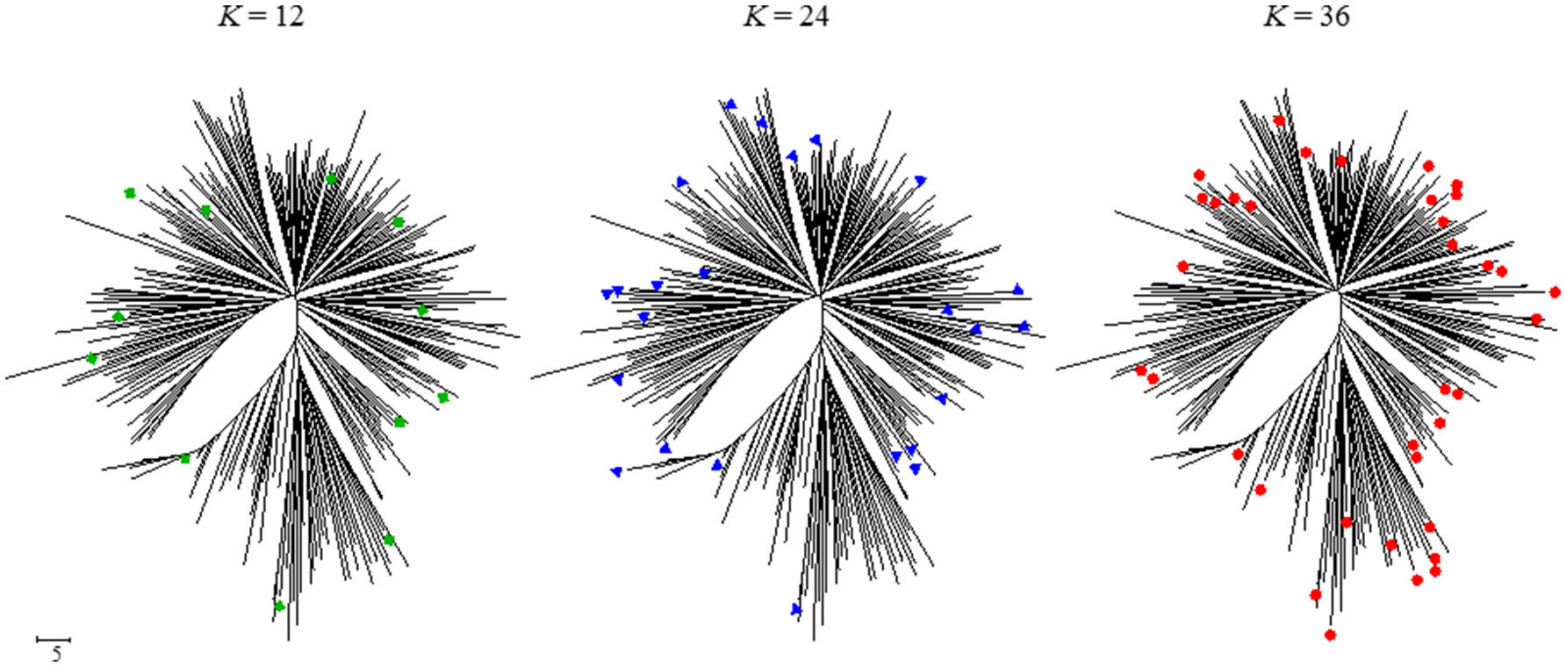
**Position on the NJ dendrogram of accessions selected for core collection (*K* = 12, *K* = 24, and *K* = 36)**.

Allele coverage (CA) reaches over 80% when CC size exceeds 36 and will continue to increase as CC becomes larger. This is explained by the large proportion of rare alleles in the total number of alleles detected (71.7% of total alleles); the larger the CC, the higher the number of rare alleles included in it. The frequency of rare alleles in CC36 was 197/352 (56.0%). Members of CC36 were evenly distributed on the dendrogram drawn by neighbor-joining method using the same dataset for CC selection. Compared with the distribution of a smaller number of the CC, CC36 members filled the non–represented branches by those CC12 and CC24 selected members (Figure 2).

The distribution patterns of the 298-original collection and CC36 were compared with geographical origin, botanical race distribution, and ratio of genetic group determined by STRUCTURE analysis. The original collection consists of accessions collected from 17 States in Mexico and 7 other countries. Elements selected in CC36 were collected from 9 Mexican States and one other country (Costa Rica), encompassing the most representative area of distribution of the geographic origin of accessions in the original collection. Components of botanical races and species in the original collection and CC36 were 104 (32.6%) and 11 (30.6%) for *drymifolia*; 15 (4.7%) and 3 (8.3%) for *guatemalensis*; 12 (3.8%) and 1 (2.8%) for *americana*; 3 (0.9%) and 2 (5.6%) for *costaricensis*; 31 (9.7%) and 3 (8.3%) for hybrids; as well as 148 (46.4%) and 14 (39%) for unknown. The ratio of genetic groups determined by STRUCTURE analysis of the original collection and CC36 were almost the same. A list of the 36 accessions is found in Supplementary Table 1.

## Discussion

### Genetic characteristics and diversity of the BNGA collection in CEBAJ-INIFAP

Mexico is the center of origin of avocado and there exist several germplasm collections and conservation activities regarding the species (Ashworth et al., 2011). However, there is a limited number of studies about genetic diversity and background of Mexican varieties. Among these studies, a global evaluation of avocado germplasm conserved in USA (Schnell et al., 2003) included 51 accessions of *drymifolia*. Cuiris-Pérez et al. (2009) used ISSR markers from 77 accessions of a nationwide avocado collection in Uruapan, Michoacan and reported high variation among varieties while focusing specifically on the Mexican race (*drymifolia*). Galindo-Tovar et al. (2011) also studied genetic relationships of avocado in Mexico using microsatellites and claimed to have found two genetic groups; however, the samples analyzed belonged to a considerably limited geographic area.

In this work, a considerable number of accessions (318 accessions) from the BNGA collection were used to illustrate the genetic diversity and background of avocado present in Mexico. As the BNGA collection in CEBAJ-INIFAP contains accessions from a wide geographical range in the country, it served as an optimal data source to infer genetic diversity and background of avocado present in Mexico. The result of this study demonstrated that this collection has achieved as much genetic diversity as other international avocado collections. Expected genetic diversity (*H*e) values of the BNGA collection (*H*e = 0.75) were smaller than those of 42 accessions of avocado varieties preserved in several institutes in Spain (*H*e = 0.831), which consist of a mixture of *drymifolia, guatemalensis*, and *americana* collected from a broad geographical range (Gross-German and Viruel, 2013). The total average of the detected number of alleles at the BNGA collection (*N*a = 18.9, Table 2) exceeds that of the Spanish collection (*A* = 11.4). This high genetic diversity of the BNGA collection has derived mainly from the presence of rare alleles, which account for 71.7% (393/548) of total alleles. These rare alleles give significant values to Mexican avocado and emphasize the importance of conservation.

### High linkage disequilibrium in avocado in Mexico

Human imposed selection, intentional, or otherwise, modifies genetic diversity as it limits the number of lineages that are maintained for propagation. Although avocado is a subtropical tree species which predominantly outcrosses, we observed a deviation from HWE as well as a significant level of LD between pairs of markers in the materials used in this study. This could be explained by the extensive number of alleles being analyzed, yet it may suggest a domestication bottleneck process experienced differently by each avocado botanical race. Use of unlinked or weakly-linked genetic regions is recommended for STRUCTURE analysis to make meaningful inferences (Falush et al., 2003). Our results, especially *drymifolia*, demonstrated LD and substantially high deviation in all loci from HWE, which should be considered while interpreting these data. Chen et al. (2008) studied LD in wild avocado by sequencing 4 nuclear loci. They reported that significant excess of interlocus LD was observed when the three botanical races (*drymifolia, guatemalensis* and *americana*) were analyzed together, but not when the analysis was performed within each botanical race. They concluded that the LD arose from the genetic structure of the different botanical races used in their study.

Although in small proportion, compared to *drymifolia* (104), our initial analysis included a small number of individuals of *guatemalensis* (15), *americana* (12), and *costarricensis* (3), as well as some non-*P. americana* species. We contrasted this analysis with one that only included *drymifolia* and no significant difference was found in this comparison (data not shown). The same approach was carried out with CC36 and, although the LD decreases at several loci, the overall high LD tendency prevailed. Microsatellite markers used in this study consisted in est-SSR and genomic SSR markers. Both markers suggested interlocus LD, and showed no evidence of selection at the est-SSR loci corresponding to the markers analyzed in this study. Currently, genome sequencing of Mexican avocado is in process (Ibarra-Laclette et al., 2015). The complete genomic information will allow us to carry out further studies regarding interlocus LD and association mapping.

### Implication of avocado domestication by population structure analysis

Previous studies succeeded in grouping based on the botanical races using genetic classification by STRUCTURE analysis (Gross-German and Viruel, 2013). In this study, STRUCTURE analysis and clustering pattern based on utilized markers suggest that there are two genetic groups within the BNGA collection that did not coincide with determination of botanical races. A previous study based on ISSR analysis also reported two genetic groups in botanical race *drymifolia* (Cuiris-Pérez et al., 2009). Chen et al. (2009) evaluated haplotype analysis by sequencing 4 nucleic genes and reported that two genetic groups, which were determined by longitude and altitude, exist in wild avocados of Mexico. Torres-Gurrola et al. (2009) evaluated foliar chemical diversity using 35 accessions conserved in BNGA in CEBAJ-INIFAP, which partly coincide with materials used in this study, but the chemical profile did not demonstrate clear correlation between geographic distributions. The grouping pattern of our study partly coincides with results of the Martínez-Villagomez et al. (2016) analysis of the distribution of genus *Persea* based on climate parameters. The distribution of our Cluster A coincides with their group I (humid semi-warm to humid semi-cold), and our Cluster B with their group II (humid semi-warm to hot semi-dry). This congruence between genetic clustering and climate distribution clustering might imply that avocado in Mexico may have evolved separately in two climatic regions, which could have also led to differences in both est- and genomic microsatellite markers.

Other than the geographical/environmental factors, this grouping may have some relation with possible multiple domestication of avocado in Mexico. Clegg et al. (1993) suggest that several domestication events for avocado occurred in the past. Gama-Campillo and Gomez-Pompa (1992) concluded that avocado is a semi-domesticated tree and is still under the process of domestication by frequent exchange of trees/seedlings between wild habitat and home garden/orchard. Very low values of divergence of allele frequencies, especially found in Cluster B (0.05), suggests that the materials used in this study have also undergone such process.

Since genetic flow persists both among cultivated botanical races and non-cultivated wild avocadoes, correlation between race-identification based on morphological characteristics (Table 1) and genotypic information is difficult to achieve in Mexican avocado. Based on their morphological characteristics, 31 accessions were preliminarily identified as hybrids between botanical races. However, results rendered by STRUCTURE analysis identified that 59 accessions possess admixed genetic background. It is known that the complexity of the hybrid status (e.g., multiple backcrossing), and segregation of a race-specific trait (e.g., green or black skin) in hybrid origin progenies have made determination of botanical assignments extremely difficult (Ashworth and Clegg, 2003).

Molecular studies in clustering analysis have provided general agreement between botanical-race classification and the employed molecular markers with some exceptions (Ashworth and Clegg, 2003; Schnell et al., 2003; Alcaraz and Hormaza, 2007; Gross-German and Viruel, 2013). However, these studies are primarily based on improved varieties that have trackable pedigree records. Our study, on the other hand, is mainly based on local cultivars in the center of origin of the species, where gene flow is to be expected among their wild counterparts. Therefore, genealogical relationships are harder to determine and may also explain why botanical races do not coincide with the selected genotype-based clustering.

### Core collection and long-term conservation of the BNGA collection

Long-term conservation of commercially important tree species is one of the most urgent and challenging strategies aimed to assure the availability of such resources for future generations. This becomes particularly difficult with recalcitrant species. In the present work, we developed a core collection from 298 genotypes conserved in the BNGA in CEBAJ-INIFAP by PCA-K means clustering method with 28 microsatellite markers.

The objective of the study was to identify a core-collection that contained genetic diversity representative of the original collection. Agro-morphological traits were excluded as a selection criterion since their inclusion would have impacted the genotypic selection process, possibly affecting the desired optimal allelic representation. Despite this exclusion, the selected CC ended up successfully representing the original collection in terms of geographical distribution, representation of botanical races, and genetic group assignment determined by STRUCTURE analysis. However, specifically designed CCs may be selected for other purposes based on different selection criteria.

Currently, the BNGA collection is maintained as field trees. This conservation method presents possible threats by natural disaster, as pest and diseases are inevitable. Although a backup conservation approach has been already implemented among field collections in different localities, such collections are still at potential risk. As an alternative, recent advances in *in vitro* and cryopreservation techniques have proven effective in safeguarding recalcitrant species with a biotechnological approach (González-Arnao et al., 2014). Cryopreservation is an efficient and less expensive alternative, becoming even more so when applied to core collections, hence the importance of an adequate CC selection process.

## Conclusion

The analysis of the BNGA avocado collection by molecular markers did not establish a clear difference among avocado botanical races. Our results suggest that the two genetic groups inferred were admixed and have contributed to the development of the current genetic structure of such populations, which might present evidence for putative environmental and/or domestication history of the species. This information, combined with agro-morphological characteristics, could prove useful when used for association studies, important in the acceleration of breeding procedures.

The selected core collection efficiently represents the diversity of the original collection and is a suitable candidate set for long-term cryopreservation conservation. This strategy may serve as a model for the conservation of other important recalcitrant species.

## Author contributions

RM conceived and designed the experiments; EH, ME, MC, and EB provided reagents/materials/analysis tools; LG and RM performed the laboratory procedures; RM, LG, EB, and ME analyzed the data; LG and RM wrote the paper; EH, ME, MC, and EB edited and provided critical review of the manuscript. All authors read and approved the final manuscript.

### Conflict of interest statement

The authors declare that the research was conducted in the absence of any commercial or financial relationships that could be construed as a potential conflict of interest.
