# Supplementary material for: Genetic Structure and Selection of a Core Collection for Long Term Conservation of Avocado in Mexico
Source: Front Plant Sci. 2017 Feb 24;8:243. doi: 10.3389/fpls.2017.00243 (PMC5323459; doi:10.3389/fpls.2017.00243)
Supplement: Supplementary file 2 [file Image1.pdf]

Supplementary Material

Genetic structure and selection of a core collection for long term conservation of avocado in Mexico

Luis Felipe Guzmán1†, Ryoko Machida-Hirano2†*, Ernesto Borrayo2, Moisés Cortés-Cruz1, María del Carmen Espíndola-Barquera3, Elena Heredia García4

*** Correspondence:** Corresponding Author: hiranoryoko@gmail.com

1 Supplementary Data

Supplementary Material should be uploaded separately on submission. Please include any supplementary data, figures and/or tables.

Supplementary material is not typeset so please ensure that all information is clearly presented, the appropriate caption is included in the file and not in the manuscript, and that the style conforms to the rest of the article.

2 Supplementary Figures and Tables

For more information on Supplementary Material and for details on the different file types accepted, please see here.

2.1 Supplementary Figures

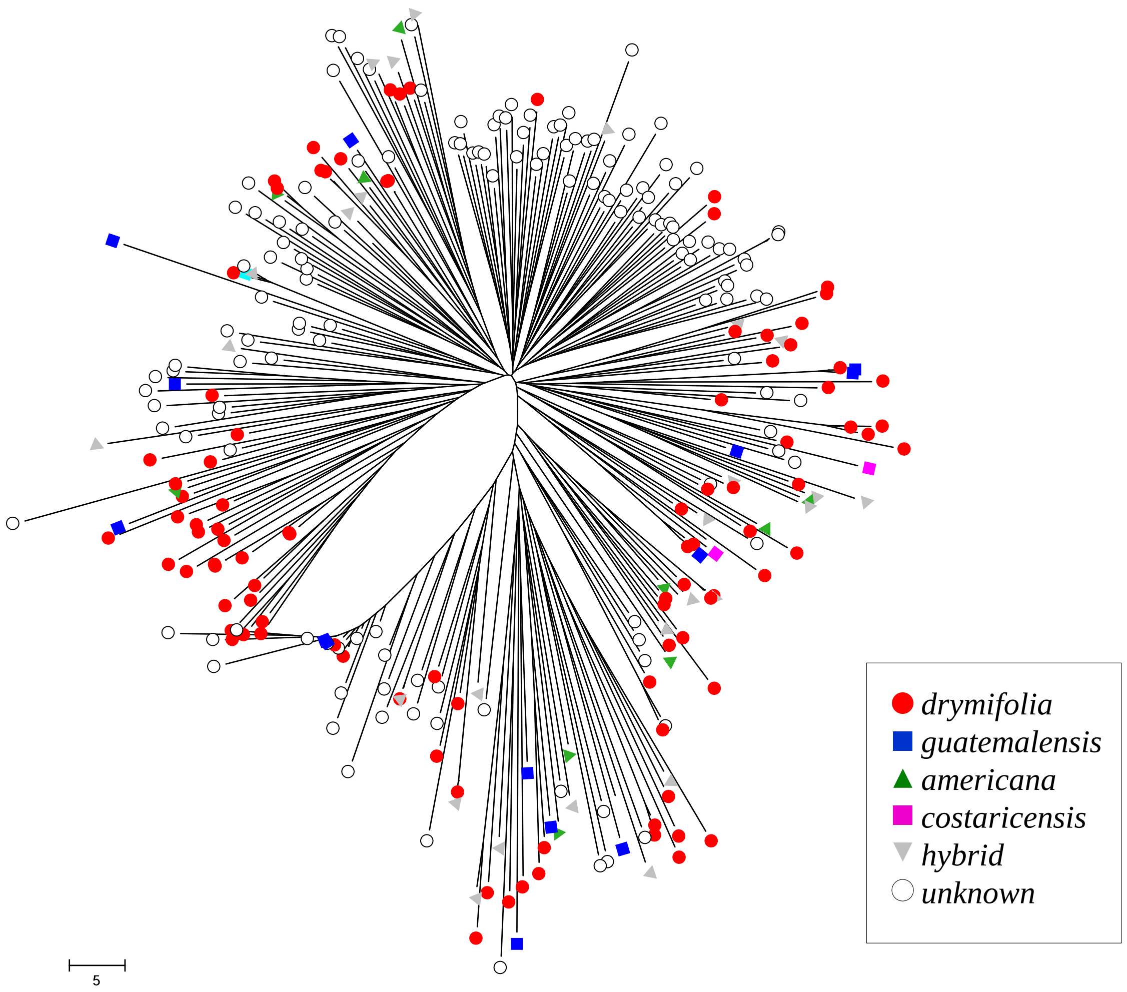

Supplementary Figure 1. Neighbor joining tree of the 298 accessions analyzed. Symbols represent botanical races of each accession.

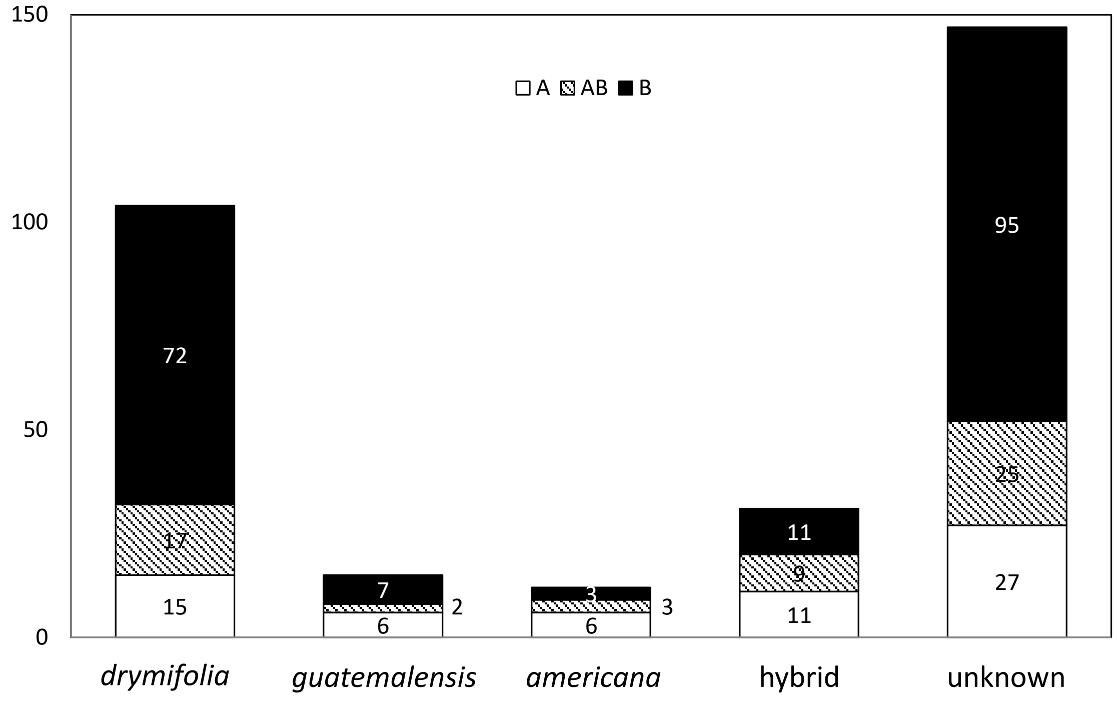

Supplementary Figure 2. Relations between botanical race and population assignment results by STRUCTURE (*K* = 2).
